# Evaluation of Commercial Myxomatosis Vaccines against Recombinant Myxoma Virus (ha-MYXV) in Iberian Hare and Wild Rabbit

**DOI:** 10.3390/vaccines10030356

**Published:** 2022-02-24

**Authors:** Fábio A. Abade dos Santos, Carina L. Carvalho, Pâmela C. L. G. Valente, Henrique Armés, Sylvia S. Reemers, Maria C. Peleteiro, Inés Calonge Sanz, Kevin P. Dalton, Francisco Parra, Margarida D. Duarte

**Affiliations:** 1 Centre for Interdisciplinary Research in Animal Health (CIISA), Faculdade de Medicina Veterinária, Universidade de Lisboa, Avenida da Universidade Técnica, 1300-477 Lisbon, Portugal; pamppam6@hotmail.com (P.C.L.G.V.); mcpelet@fmv.ulisboa.pt (M.C.P.); margarida.duarte@iniav.pt (M.D.D.); 2 Instituto Nacional de Investigação Agrária e Veterinária (INIAV, I.P.), Av. da República, Quinta do Marquês, 2780-157 Oeiras, Portugal; carina.carvalho@iniav.pt; 3 Instituto Universitario de Biotecnología de Asturias (IUBA), Departamento de Bioquímica y Biología Molecular, Universidad de Oviedo, 33006 Oviedo, Spain; uo280761@uniovi.es (I.C.S.); daltonkevin@uniovi.es (K.P.D.); fparra@uniovi.es (F.P.); 4 Associate Laboratory for Animal and Veterinary Sciences (AL4AnimalS), 1300-477 Lisbon, Portugal; 5 Hospital Veterinário de São Bento, R. de São Bento 358a, 1200-822 Lisbon, Portugal; henrique.armes@veterinario.pt; 6 MSD Animal Health, Wim de Körverstraat 35, 5831 AN Boxmeer, The Netherlands; sylvia.reemers@merck.com

**Keywords:** myxomatosis, Iberian hare, European wild rabbit, vaccines, ha-MYXV

## Abstract

The recent emergence of a new myxoma virus capable of causing disease in the Iberian hare (*Lepus granatensis*) has resulted in numerous outbreaks with high mortality leading to the reduction, or even the disappearance, of many local populations of this wild species in the Iberian Peninsula. Currently, the available vaccines that prevent myxomatosis in domestic rabbits caused by classic strains of myxoma virus have not been assessed for use in Iberian hares. The main objective of this study was to evaluate the efficacy of commercial rabbit vaccines in Iberian hares and wild rabbits against the natural recombinant myxoma virus (ha-MYXV), bearing in mind its application in specific scenarios where capture is possible, such as genetic reserves. The study used a limited number of animals (pilot study), 15 Iberian hares and 10 wild rabbits. Hares were vaccinated with Mixohipra-FSA vaccine (Hipra) and Mixohipra-H vaccine (Hipra) using two different doses, and rabbits were vaccinated with the Mixohipra-H vaccine or the Nobivac Myxo-RHD PLUS (MSD Animal Health) using the recommended doses for domestic rabbits. After the vaccination trials, the animals were challenged with a wild type strain of ha-MYXV. The results showed that no protection to ha-MYXV challenge was afforded when a commercial dose of Mixohipra-FSA or Mixohipra-H vaccine was used in hares. However, the application of a higher dose of Mixohipra-FSA vaccine may induce protection and could possibly be used to counteract the accelerated decrease of wild hare populations due to ha-MYXV emergence. The two commercial vaccines (Mixohipra-H and Nobivac Myxo-RHD PLUS) tested in wild rabbits were fully protective against ha-MYXV infection. This knowledge gives more insights into ha-MYXV management in hares and rabbits and emphasises the importance of developing a vaccine capable of protecting wild populations of Iberian hare and wild rabbit towards MYXV and ha-MYXV strains.

## 1. Introduction

In 2018, a natural recombinant myxoma virus (referred to as ha-MYXV or MYXV-Tol08/18) emerged in the Iberian hare (*Lepus granatensis*), affecting many populations in Spain [1,2] and Portugal [3,4,5].

With an apparent mortality rate of 55.4% [6], the geographic spread of ha-MYXV outbreaks increased concerns about the resilience limit of the Iberian hare wild populations against the many insidious factors that have accelerated their decline over the last decades [5]. Despite the conservation status of the Iberian hare (*Lepus granatensis*) being considered of “Least Concern” by the IUCN in 2019, many local populations are currently threatened, as a result of severe loss of habitat imposed by human activities, and more recently, due to the emergence of infectious diseases such as ha-MYXV and LeHV-5 [2,3,4,5] and the cumulative effects with other diseases such as cysticercosis [5].

Although ha-MYXV was initially detected only in Iberian hares, it was later (mid-2020) reported in wild and domestic rabbits [7,8]. The recognition that ha-MYXV affects not only hares, but also the European rabbit (*Oryctolagus cuniculus*), questions the efficacy of cross-protection conferred by classic field strains of MYXV that circulate in wild rabbits. Likewise, the effectiveness of the commercial vaccines developed to protect domestic rabbits against classic myxoma strains is still unknown with regard to infection with ha-MYXV.

The susceptibility of the European rabbit to ha-MYXV also escalated the previous concerns that in 2019 gave the wild European rabbit (*Oryctolagus cuniculus*), for the first time in history, the status of “Endangered of Extinction”, by the IUCN [9,10].

Several strategies have been attempted to protect and recover the native wild rabbit populations for conservation and hunting purposes, such as captive breeding, re-introduction, restocking programs and vaccination campaigns, reviewed in [11].

This analysis constitutes a pilot study using a small number of wild animals to assess the commercial vaccines Mixohipra-FSA and Mixohipra-H as prophylactic tools to protect Iberian hare against ha-MYXV, bearing in mind its use in captive populations. The present study also evaluated the protection conferred by commercial vaccines Mixohipra-H and Nobivac Myxo-RHD PLUS against ha-MYXV in wild European rabbit. The possibility of using these vaccines as prophylactic tools in wild leporids whenever possible may be crucial to ensure the preservation of the genetic viability of the species in Portugal and Spain, particularly in case of an aggravation of the actual sanitary situation. This study also intends to test the susceptibility of wild rabbit to ha-MYXV isolated from the Iberian hare.

This study puts into practice the Measure 7.6 of Project + Coelho 2, entitled “Evaluation of the efficacy of commercial vaccines against myxomatosis in Iberian hare”, identified within the National Plan for the control of Rabbit Haemorrhagic Disease 2 in rabbits (Dispatch 4757/17, 31 may ordered by the Minister of Agriculture), as it is vital to test all available resources in the fight against this emergent virus. This study, therefore, constitutes a pivotal step by assessing the potential of commercially available vaccines for the protection of wild Iberian hare and European rabbit.

## 2. Materials and Methods

### 2.1. Origin of Animals and Pre-Adaptation to Captivity Conditions

The main study (study 1) used nine 9-month-old, MYXV antibody seronegative, male hares (*Lepus granatensis*) randomly divided into 3 groups. These animals were the first generation born in captivity and were maintained in semi-extensive conditions for the sole purpose of this study. Their progenitors were captured in South Portugal between 31 August 2019 and 9 January 2020. Capture and accommodation of the hares were approved by the Institute for the Conservation of Nature and Forests (ICNF, I.P.), the Portuguese National Authority for Nature Conservation. A MYXV-seropositive, two-year old adult male hare, was used as control of the natural humoral response efficacy against the ha-MYXV.

A total of 10 wild rabbits (*Oryctolagus cuniculus algirus*), 6 months of age, seronegative for antibodies against MYXV were used in this study (study 2). The animals were obtained from a wild rabbit reproduction farm, where they were kept in captivity under extensive farming (approved by the National Authority (ICNF, I.P.).

Separately (study 3), after the conclusion of studies 1 and 2, an additional group of five 5-month-old hares, MYXV-seronegative, born in captivity after the start of the first trial, 3 females (#013, #014 and #020) and 2 males (#021 and #025), were used to further confirm the results obtained in study 1.

To avoid the effects of captivity stress on the immune response triggered by vaccination, the animals were adapted through a 40-day quarantine in specifically designed and constructed cages (structure description available upon request). These species-specific cages had different dimensions and particularities to allow the expression of natural hare behaviour (grooming, station position, etc.) as much as possible, to minimize injuries and to allow safe handling during the introduction and removal of the animal from the cage. The hare cages included an additional closed, opaque, confined space, to provide refuge and allow the animals to remain quiet.

A subjective rating of the animals’ behaviour was scaled from 1 to 3. A rating of 1 indicates a reduced reluctance to handling and sample collection, a rating of 2 indicates a reluctance to initial handling but an absence of sudden movements and a rating of 3 indicates reluctance throughout the entire procedure with many sudden movements.

The animals were observed daily for food, water intake and behaviour through uninterrupted monitoring cameras to assess the signs of disease or stress. The rabbit watering and feeding system did not allow the individual quantitative analysis of drinking water and daily food intake. The animals were acquainted with the staff (4 veterinarians) that carried out the trial. All the animals were subjected to hemogram and biochemical tests, faecal and blood parasite analyses before the study. The animals were considered fit for study when no signs of stress such as weight loss or signs of illness were observed and normal behaviour was maintained.

### 2.2. Vaccines

Three commercial vaccines against myxomatosis were used: (i) Mixohipra-FSA (HIPRA Headquarters, Amer, Girona, Spain; lot 12M9J), a live heterologous attapulgite adjuvanted vaccine containing Shope fibroma virus; (ii) Mixohipra-H (HIPRA Headquarters, Amer, Girona, Spain; lot 05D7G), a live homologous vaccine containing attenuated myxoma virus; (iii) Nobivac Myxo-RHD PLUS (MSD Animal Health, Boxmeer, The Netherlands; lot A003B02), a live homologous vector vaccine containing two attenuated recombinant myxoma virus vectors expressing the VP60 gene of RHDV or RHDV2 [12].

The investigational veterinary products (IVP) were used within the stated shelf-life.

### 2.3. Experimental Design

The hares of study 1 were randomly divided into four groups and identified with an earring according to the following:

(i) H-G1: a negative control group of three animals (#231, #232 and #233), MYXV seronegative and non-vaccinated;
(ii) H-G2: a group of three animals (#076, #077 and #078), MYXV seronegative, vaccinated with a 2.90 × 10^4^ ffu (focus-forming units) dose of Mixohipra-FSA vaccine at the start of the study and 21 days later with a 1.95 × 10^4^ ffu dose of Mixohipra-H vaccine;
(iii) H-G3: a group of three animals (#042, #043 and #044), MYXV seronegative, vaccinated with a 2.90 × 10^5^ ffu dose of Mixohipra-FSA vaccine at the start of the study and 21 days later a 1.95 × 10^5^ dose of Mixohipra-H vaccine;
(iv) H-G4: a positive control animal (#10), MYXV seropositive, collected from the field after natural recovery from myxomatosis.

The wild rabbits (study 2) were randomly divided into three groups and identified with an earring according to the following:

(i) R-G1: a negative control group of four animals (#449, #451, #000 and #001), MYXV seronegative and non-vaccinated;
(ii) R-G2: a group of three animals (#442, #444 and #445), MYXV seronegative, vaccinated with a 1.95 × 10^4^ ffu dose of Mixohipra-H vaccine at the start of the study;
(iii) R-G3: a group of three animals (#446, #447 and #448), MYXV seronegative, vaccinated with a single dose of Nobivac Myxo-RHD PLUS vaccine (10^3.0^–10^5.8^ ffu of each two vectors) at the start of the study.

**Figure 1:**
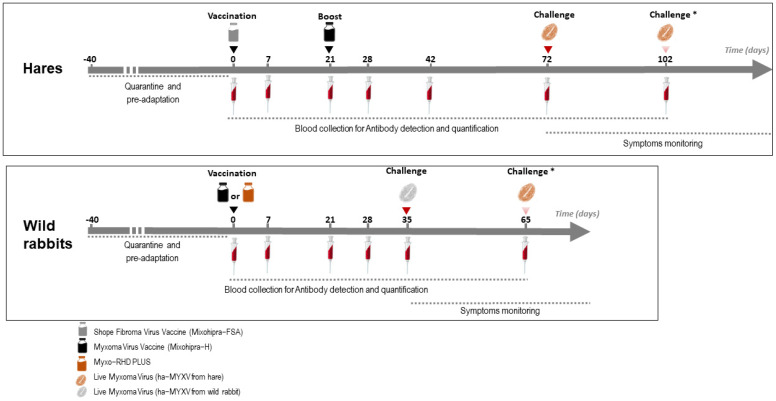
Schematic representation of the vaccination and sampling schedule performed on hares and rabbits (Studies 1 and 2). * The second challenge was only carried out on part of the animals (see Section 2.8).

A schematic overview of the trials is depicted in Figure 1. The vaccines were inoculated subcutaneously (s.c.) with the volume of the vaccine distributed in two different places in the dorsal cervical area. At the time of vaccination, the groups of non-vaccinated animals (#H-G1, H-G4 and R-G1) were inoculated s.c. with 1 mL of sterile phosphate-buffered saline (PBS) pH 7.2.

The additional group of five 5-month-old hares (study 3) was vaccinated as group H-G3, with an infectious dose of 2.90 × 10^5^ ffu of Mixohipra-FSA vaccine and 21 days later revaccinated with Mixohipra-FSA vaccine with the same infectious dose (2.90 × 10^5^ ffu).

The hare and rabbit experimental trials were carried out separately. Between experiments, the installations were cleaned and disinfected, submitted to 24 h of UVC irradiation, and subjected to a 60-day sanitary vacuum. During these two trials, blood samples were collected from all animals according to the protocol represented in Figure 1. At the time of sampling, body weight, respiratory rate and rectal temperature were also monitored.

### 2.4. Sampling and Blood Analyses

Given the nervous natural behaviour of wild leporids, wilder in the Iberian hare than in the wild rabbit, there is a high risk of self-injury during handling, especially affecting the lumbar spine following sudden movements in reaction to human presence or restraint. For this reason, the collection of samples was minimized to avoid the impact of stress on the results and to reduce the risk of harming the animals during the procedures.

The animals were handled with slow but assertive movements without sedation or anaesthesia. Blood collections were performed according to the method described by [13], without midazolam administration to avoid the effects of recurrent sedation.

The volume of blood collected from hares and rabbits was 6 mL and 1 mL, respectively. The blood was divided into three collection tubes, namely dry for serology, EDTA for hemogram and lithium heparin for biochemical analyses.

On the same day of sampling, serum was separated from the clot by centrifugation at 1000× *g* for 10 min at 4 °C. The haematological analyses were performed automatically using the Procyte Dx haematological counter (IDEXX^®^) with further manual correction. The haematocrit was confirmed and corrected by the evaluation of the microhematocrit tube.

The relative count of leukocytes and the search for haemoparasites were performed by microscopical examination (400–1000× magnification) of blood and buffy coat smears stained with Diff-Quik.

Biochemical analyses were performed in a Catalyst One Chemistry Analyzer (IDEXX, Westbrook, ME, USA) using the Chem 15 CLIP consumables. All the analyses were performed in duplicate.

### 2.5. Serological Analyses

Sera were analysed for MYXV antibodies using a commercial indirect ELISA (iELISA) (Civtest^®^ Cuni Mixomatosis, Hipra, Girona, Spain), following the manufacturer’s instructions. Positive and negative controls (rabbit sera, provided in the kit) and samples were tested in duplicate. Results were expressed as Relative Index 10 (RI10).

All sera were also analysed by an in house immunofluorescence test (IFT) [7] using the intervals previously described [12].

The titre of the immunofluorescence test was estimated using 4 replicates for each dilution. Some hare sera were additionally tested at the OIE International Reference Laboratory of Myxomatosis (IZLER, Brescia, Italy), using a competitive ELISA (cELISA).

The titre of the seroneutralization test was estimated using 2 replicates for each dilution (method available upon request). Briefly, sera were initially inactivated by incubation at 56 °C for 30 min. Sera were then two-fold diluted from 1/4 to 1/2048 and incubated 2 h at 37 °C with 100 ffu of ha-MYXV (strain 20545PT20) using a 96-well plate. Then, 0.01 × 10^6^ RK13 cells were sown in Gibco MEM medium (Thermo Fisher Scientific, Waltham, MA, USA) with 5% heat-inactivated foetal bovine serum—FBS (Thermo Fisher Scientific, Waltham, MA, USA). Virus plaques were visualized after 5 days of incubation and the titre considered the mean of the last dilutions that inactivated 100% of the virus.

### 2.6. Viruses

Viral isolation of ha-MYXV from eyelid samples from an infected wild rabbit (20545PT20, found dead in July 2020, Portugal) and from an infected hare (38455PT18, found dead in November 2018 in Portugal) were carried out using Rabbit Kidney (RK13) cells (ATCC-CCL-37). The rabbit and hare ha-MYXV strains were adapted to cell culture by seven passages, and then purified and titrated.

In detail, tissue samples were homogenised at 20% (*w*/*v*) in PBS containing penicillin, streptomycin and amphotericin B (antibiotic-antimycotic), used according to the manufacturer (Gibco, Waltham, MA, USA). Following centrifugation (3000× *g*, 10 min, 4 °C), the supernatant was filtered through a 0.45-µm-pore-size filter (Millipore Express, Darmstad, Germany) and used to inoculate subconfluent (70%) RK13 cells grown in Eagle’s medium supplemented with 5% FBS, penicillin, streptomycin and amphotericin B (antibiotic-antimycotic used at 1:100, Gibco) and 50 µg/mL gentamicin (Gibco). Cells were maintained at 37 °C in a humidified atmosphere with 5% CO2 and observed daily for cytopathic effect (CPE) by phase-contrast microscopy. Virus isolation was confirmed by an in-house IFT using MYXV positive hare serum [7]. Virus isolates were named 20545PT20 and 38455PT18, according to the identification of origin, and used for the immunofluorescence tests and seroneutralization assays.

Unlike the virus used in the IFT, produced as described above, the viruses used in challenge experiments were not subjected to cell culture passages to avoid mutations and recombination events during in vitro culturing, and correspond to the same isolates (20545PT20 and 38455PT18). The oedematous eyelids removed from these animals were washed seven times with sterile PBS pH 7.2 with mechanical agitation, to remove external tissue contaminants resulting from the accumulation of purulent material during the illness. After initial scalpel blade fragmentation, a 10% (*w*/*v*) tissue homogenate was prepared in sterile PBS pH 7.2, by mechanical maceration with 0.5 mm zirconia beads using four cycles of 15 s at 3000 rpm with an interval of 10 s (Precellys^®^ Evolution). Before and after the process, the macerates were kept on ice.

After this initial process, the viruses were purified by centrifugation using a 36% sucrose cushion and then using a 24–40% sucrose step gradient [14]. The dilutions of the virus stock for the challenge were performed using sterile PBS pH 7.2. The final batch (corresponding to the dilution inoculated into the animals) was filtered using 0.45 µm pore filters and a sample submitted to incubation in blood agar, TSA-Tryptic Soy Agar and Sahoraud’s dextrose agar, targeted to fungal agents according to routine methodology, to confirm the absence of contamination.

For stock titration, the virus was diluted in MEM (10-1 to 10-9) and adsorbed to 70% confluent RK13 cells, for 1 h at 37 °C. After 5 days of incubation, plates were washed with PBS and fixed with 70% Acetone (Scharlab, Barcelone, Spain), for 15 min at room temperature. Then, the wells were washed with PBS and stained with 0.4% crystal violet (Sigma-Aldrich, St. Louis, MO, USA), and visualized using an inverted microscope. The titre was estimated using 4 replicates for each dilution according to a published method [15]. The ha-MYXV stocks were then diluted in sterile PBS pH 7.2 to a concentration of 100 ffu/mL (focus-forming units per millilitre).

A similar method was used to titrate the vaccine stock (only for Mixohipra vaccines), using 100 μL of reconstituted vaccine to infect 70% confluent RK13 cells in 48-well plates. The reconstituted vaccine was 10-fold diluted using MEM until 10^−6^. The vaccine virus was adsorbed for 1 h at 37 °C, and the titre was calculated after 5 days of incubation according to the previously described method [15].

### 2.7. Virus Detection by qPCR

Detection of ha-MYXV DNA by qPCR was used to confirm the isolation in RK13 cells [16]. The same molecular method was used to investigate the presence of the virus in faeces of the animals after vaccination and challenge, in tissues from the animals that died during the experiment, as well as in the drinking water. The presence of LeHV-5 was also analysed [4,17].

For nucleic acid extraction, cell supernatants or water samples were used directly for extraction, without dilution. Faeces or fresh samples of liver and spleen, kidney, lung, eyelid and genitalia were homogenised at 20% (*w*/*v*) with PBS and clarified at 3000 g for 5 min at 4 °C. Total DNA and RNA were extracted from 200 µL of the clarified supernatants, using the MagAttract 96 cador Pathogen Kit (Qiagen, Hilden, Germany) in a BioSprint 96 nucleic acid extractor (Qiagen, Hilden, Germany), according to the manufacturer’s protocol.

Amplification reactions were performed in a Bio-Rad CFX96™ Thermal Cycler (Bio-Rad Laboratories Srl, Redmond, WA, USA), using the Multiplex PCR NZYTaq 2× Colourless Master Mix (NZYTech, Lisbon, Portugal).

### 2.8. Challenge

Hares (groups H-G1, HG-2, HG-3 and H-G4) were challenged at day 72 with the isolate ha-MYXV 38455PT18. Challenge of hares was carried out by inoculating 1 mL of the virus suspension subcutaneously (100 ffu/mL of ha-MXYV, diluted in sterile PBS pH 7.2), corresponding to the maximum estimated viral load delivered by arthropod vectors in nature [18]. In case of failure to develop disease and humoral response, hares were re-inoculated with 1 mL of 1000 ffu/mL (ha-MXYV, isolate 38455PT18) at day 102 (Figure 1). Hares of study 3 were not submitted to challenge.

Wild rabbits (half of group R-G1 and groups R-G2 and R-G3) were challenged at day 35 with the isolate ha-MYXV 20545PT20 (Figure 1). Two of the negative controls (from R-G1, #000 and #001) were kept separately and were not inoculated. Thirty days later (day 65), these two rabbits were inoculated with the isolate ha-MYXV 38455PT18, the same used in the hare’s assay. The rabbits challenge was performed with 1 mL of a 1000 ffu/mLdiluted in sterile PBS pH 7.2, inoculated subcutaneously, given the failure to induce disease in two hares when inoculated with 1 mL of a 100 ffu/mL virus dilution, as discussed below.

### 2.9. Clinical Signs Monitoring

During the vaccination trial and after challenge, the animals were continuously monitored by cameras, using black light for night vision. Daily visits to the installations by a veterinarian complemented surveillance. The cages allowed us to observe the animals without handling and to photograph the evolution of clinical signs. The cages also allowed us to touch the animals without the need to hold them, allowing us to assess the health and nutritional status as well as the presence of inflammatory lesions, for example at the site of vaccine inoculation, without disturbing the animals.

To follow the evolution of clinical signs after challenge, the animals’ eyes were photographed daily. The hares’ genitals were not monitored daily to minimize stress and risk of injuries, given the need to handle the animals. However, the daily monitoring of genitals in rabbits was carried out given their easier behaviour and due to the cage characteristics.

Sequential photographs of eyelid oedema were evaluated for each animal and subjectively classified (very mild, mild, moderate and marked). The palpebral fissure height was estimated and divided into four different categories (<25%, 25–50%, 50–75% and >75%).

### 2.10. Necropsy and Histopathology

Necropsy was performed according to routine procedures, and samples were collected for bacteriology (liver, spleen and lung), parasitology (gastrointestinal tract and liver), histopathology (lung, liver, spleen, kidney, eyelid and genitalia) and virology (liver, spleen, lung, kidney, eyelid, lip, urine, seminal vesicle, brain, bone marrow, spinal cord and genitalia) analyses, following the routine procedures. For histopathology, the fragments were fixated in 10% neutral buffered formalin (*w*/*v*), routinely paraffin-embedded, sectioned at 4 µm, and stained with Hematoxylin and Eosin (H&E).

### 2.11. Ethical and Legal Framework

The study was carried out in line with the measures identified in the National Plan for the control of rabbit haemorrhagic disease 2 (Dispatch 4757/2017, 31 May), operated through Project +Coelho 2, and approved by the National Authority for Animal Health (DGAV, Nr 79/ECVPT/20145) according to the National legislation (Decree-Law No. 113/2013, 7 August) after a positive declaration from the independent Advisory Body Responsible for Animal Welfare (ORBEA—INIAV, I.P.).

The Iberian hare specimens used in the experiment were from a pilot genetic reserve, established in 2019 within the scope of the +Coelho 2 Project, approved by ICNF. The Iberian hare founder population was captured in the field through events authorized by ICNF for the current proposal. The 14 hares selected for the study constitute the first generation born in captivity. The seropositive hare (#10) was captured in the field in October 2019. All wild rabbits used in this study were purchased from a certified wild rabbit captivity centre complying with the Portuguese legislation. Vaccination and challenge were conducted in a BSL-2 mobile unit belonging to INIAV I.P.

Taking into account the 3R policy, all steps of this assay that could be performed in vitro were maximized (replacement) (e.g., isolation, virus multiplication), reducing the number of animals to the minimum likely to give results for a pilot study (reduction) and all manipulation techniques, data analysis and maintenance of the animals, were thought and designed specifically (refinement) for the Iberian hare and wild rabbit (e.g., BSL-2 built specifically for the study, specific cages were built and adapted to the needs of these two species. To minimize stress and self-inflicted damage, the animals were always handled by the same people and kept in a calm environment, etc.)

## 3. Results

**Figure 2:**
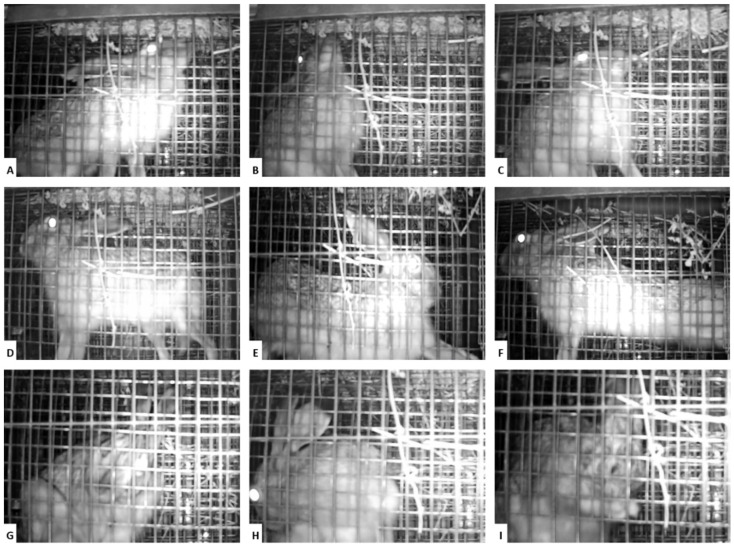
Pictures from the continuous monitoring performed by backlight cameras. The cages were designed to provide cognitive enrichment, making the access to alfalfa hay difficult (**A**–**C**), to allow all the hare’s natural positions, namely muscle stretching (**D**–**F**). Animal welfare can be observed, among other factors, by the presence of grooming, which is very common in this species (**G**–**I**).

The data described below refer to the quarantine and vaccination periods (from day −40 to day 72 in the hare trial and until day 35 in the rabbits’ trial), i.e., before the challenge. Surveillance through day and night viewing cameras revealed an adequate adaptation to cages, expression of natural behaviour (Figure 2) and maintenance of water and food ingestion within the normal range.

The 10 hares entered study 1 with an average weight of 1.90 ± 0.26 kg. All weight measurements were carried out in the morning, at approximately the same time. The maximum loss of weight before the challenge was 5.55%. Behaviour was stable throughout the entire experiment, with only a slight deterioration over the number of handlings, starting with an average rating of 1.3 in the first sampling and ending with 2.1 in the last one (after seven sampling procedures). The average respiratory frequency after the initial containment was 87 ± 13 breaths per minute (bpm) at the moment of first sampling and of 108 ± 9 bpm at the moment of the last sampling. The temperature was also measured in all sampling moments, showing an average of 39.5 ± 0.4 °C.

The rabbits did not have any remarkable loss in weight; on the contrary, they gained weight throughout the experiment (an average of 10.3%), the mean weight before the challenge being 1.12 ± 0.27 kg. The rabbits had an average temperature of 39.05 ± 0.1 °C in the five moments of blood sampling and a mean breath rate of 58 ± 23 bpm. The behaviour of rabbits was substantially more favourable compared to hares, starting with an average rating of 1 in the first sampling and ending with 1.2 in the last (after five sampling events). In both species, the variations in weight, respiratory rate and rectal temperature were considered physiological, since the start of the study until the start of the challenge.

### 3.1. Humoral Immune Response to Vaccination

In general, the hares vaccinated with the lower dose of the two Mixohipra vaccines (H-G2), did not produce a serological response, except for hare #077, which achieved an RI10 title of 2.81. However, this hare died suddenly on day 51, hampering the follow-up of the next phases of the experiment. Necropsy showed that the cause of death of hare #077 was dysbiosis, probably due to stress, the animal being in good body condition.

Two of the three hares vaccinated with the higher dose of the two Mixohipra vaccines (H-G3), seroconverted, achieving an RI10 value of 5.8 (hare #043) and 7.1 (hare #044) by day 72 of the trial. Hare #042 showed no seroconversion during the experiment. The increase in serological response was higher after inoculation with the Mixohipra-FSA vaccine, namely between day 0 to day 21. The increase in serological response after boost vaccination with Mixohipra-H vaccine, between day 21 and day 42, was much lower or even absent (Table 1). By day 21, hares #043 and #044 registered a titre of 10 and 20, respectively, in the cELISA, while all the other hares remained negative. These titres remained unchanged, even after the boost vaccination.

These results were further confirmed in study 3, with all the five hares seroconverting. At day 0, the RI10 was < 1.0 in the five animals and 21 days later was 4.6, 4.9, 3.7, 8.2 and 4.9 for hares #013, #014, #020, #021 and #025, respectively. Twenty-one days after the second vaccination, the RI10 values obtained with the iELISA were 6.7, 6.6, 7.5, 11.2 and 5.4, respectively. Contrary to animals #043 and #044 (H-G3), which were boosted with Mixohipra-H, the administration of a boost with Mixohipra-FSA had a positive effect on the antibody titres.

As expected, the animals from the unvaccinated control group (H-G1) did not seroconvert (Table 1).

Rabbits #448, #446, and #447, vaccinated with Myxo-RHD PLUS (R-G3) seroconverted and on day 35 achieved an RI10 titre of 2.1, 4.8, and 5.4, respectively (Table 2). Rabbits #444, #445, and #442, vaccinated with Mixohipra-H (R-G2) seroconverted and on day 35 achieved an RI10 titre of 1.72, 2.15 and 4.11, respectively. As expected, none of the animals from the non-vaccinated group, R-G1 (#449, # 451, #000 and #001) developed anti-MYXV antibodies.

### 3.2. Hematologic and Biochemical Analyses of Blood Samples Obtained during Vaccination

Monitoring of the different parameters analysed in the hemogram (RBC, HCT, HGB, MCV, MCH, MCHC, RDW, reticulocytes, WBC, neutrophils, lymphocytes, monocytes, eosinophils, basophils, platelets, MPV, PDW, PCT) showed no remarkable changes after vaccination in any of the hares and rabbits. Likewise, regarding the biochemistry analyses, no remarkable variations in glucose, creatinine, blood urea nitrogen, ALT, ALKP or GGT values were observed. Interestingly, the albumin/globulins ratio, in the case of hare #043 and hare #044 (both from H-G3), showed a decrease between days 21 and 28 after the first vaccination, coinciding with the seroconversion.

By day 30 after the challenge, sick hares showed lower haematocrit of 35.33 ± 4.51% (compared to 53.44 ± 3.24% in the healthy animals) and a leucocytosis of 18.82 ± 4.86 K/μL (WBC) (compared to 5.65 ± 1.54 K/μL (WBC) in the healthy hares), mainly resulting from a neutrophilia of 15.30 ± 7.32 K/μL (compared to 2.53 ± 1.33 K/μL in the healthy hares).

### 3.3. Clinical Course after Challenge

Hares #233 (H-G1) and #042 (H-G3) did not develop any signs of disease (Table 3) after the first virus inoculation of 1 mL (100 ffu/mL, subcutaneously), and no virus was detected in the blood, stool or conjunctival swab on day 15. Additionally, these hares did not seroconvert and were inoculated a second time, 30 days after the first virus challenge, with the same isolate, but a 10-fold higher dose (1 mL of 1000 ffu/mL). Both died after developing signs of myxomatosis (Table 3).

**Figure 3:**
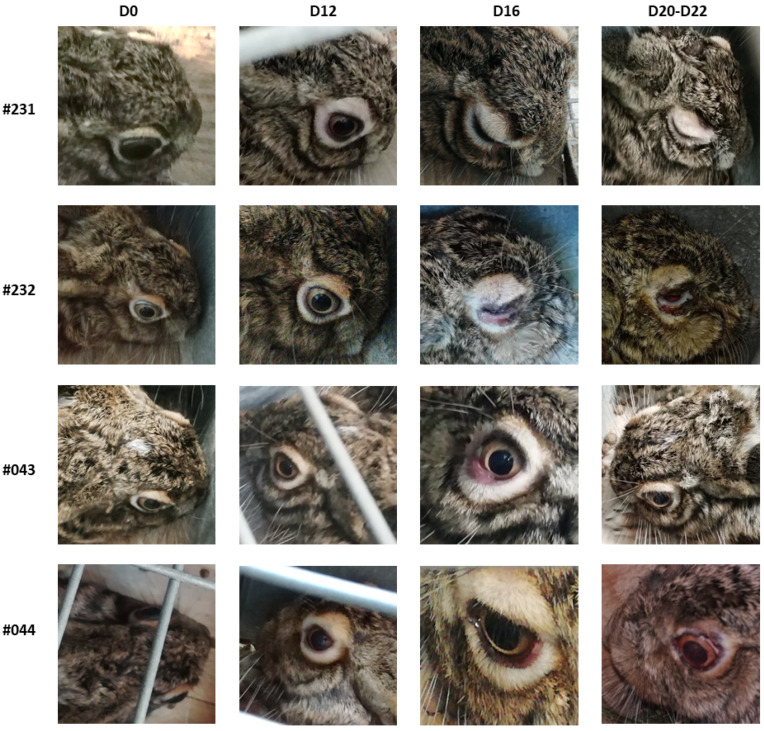
Clinical signs of myxomatosis of four hares involved in the study. Hares #231 and #232 (H-G1) were not vaccinated. Hares #043 and #044 belonged to the H-G3 group (vaccinated with 2.90 × 10^5^ ffu dose of Mixohipra-FSA). D-day after challenge.

The average incubation period of myxomatosis in hares was 11.3 ± 2.6 days considering the 8 hares, and 11.8 ± 2.2 excluding the two seropositive hares (#043 and #044), taking as a clinical reference the alterations developed in the eyelids (Table 3 and Figure 3). The evolution of oedema in the genitals was not monitored, given the difficulty of handling the sick animals and the stress effect of such handling. From the onset of symptoms, it took an average of 17.8 ± 8.5 days until death occurred or euthanasia was carried out for animal welfare purposes. Hares #043 and #044 recovered totally, respectively, in 13 and 19 days after the challenge. Hares of study 3 were not submitted to challenge.

Hares #233 (from H-G1, not vaccinated), #078 (from H-G2, vaccinated with low dose of Mixohipra-FSA and Mixohipra-H) and #042 (from H-G3, vaccinated with a high dose of Mixohipra-FSA and Mixohipra-H but challenged twice) developed severe myxomatosis and died. These hares, like those euthanized after developing severe myxomatosis (hares #231 and #232 not vaccinated (H-G1) and hare #076 vaccinated with low dose (H-G2)) lost on average 483.3 ± 147.2 g of weight from the day of virus challenge to the day of death (Table 3). In contrast, hares that developed light and shorter forms of the disease (hares #043 and #044 vaccinated with high dose (H-G3)) lost at the most 116.7 ± 28.9 g (Table 3). Comparing the hares that became very sick (>50% eyelid closed) with healthy ones, food intake decreased from 170–200 g of oats per day to 40–50% less at maximum. The opposite happened with water intake that rose from 50–70 mL to 80–110 mL per day at maximum.

**Figure 4:**
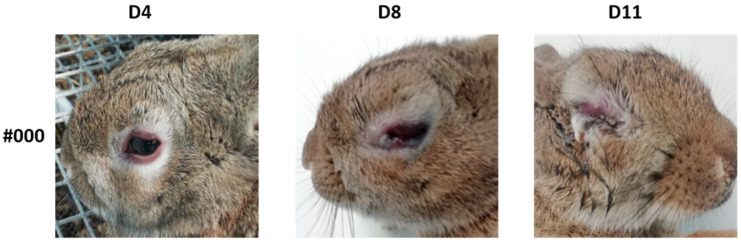
The clinical course of rabbit #000 (non-vaccinated, belonging to group R-G1) inoculated with virus isolated from Iberian hare (1 mL of 1000 ffu/mL, 38455PT18). D-day after challenge. Note very mild (D4), moderate (D8) and (D11) marked eyelid oedema.

The average incubation period of myxomatosis in positive control rabbits (R-G1, not vaccinated) was 8 days for the rabbits (#449 and #451) inoculated with isolate 20545PT20 (isolated from a wild rabbit) and 8 days for the rabbits #000 and #001 inoculated with isolate 38455PT18 (isolated from an Iberian hare) considering the alterations in the eyelids (oedema and eye closure) as reference (Table 4 and Figure 4), corresponding to an average of 6.3 ± 2.1 days in the four rabbits.

In rabbits, it took an average of 5.25 ± 1.5 days from the onset of symptoms until death occurred, and an average of 11.5 ± 0.56 days from the virus challenge until death, with an average weight loss of 73.5 ± 45.0 g. None of the vaccinated rabbits showed symptoms of myxomatosis (Table 4).

Interestingly, the incubation period among rabbits inoculated with wild rabbit virus isolate (20545PT20) was longer (8 days) compared to Iberian hare virus isolate (38455PT20) (4.5 days). After the onset of symptoms, rabbits inoculated with strain 20545PT20 died within 4 days and rabbits inoculated with a strain of Iberian hare died within 6.5 days. As expected, rabbits with a longer course of disease showed more severe pathological signs (Table 4).

The MYXV antibodies estimated by iELISA at the death or recovery moments revealed RI10 values of 9.12, 1.82 and 0.23 for the #231, #232, #233 (H-G1, nonvaccinated); 2.62 and 0.12 for #076, #078 (H-G2, vaccinated with the lower dose); and 0.23, 31.00 and 28.90 for #042, #043 and #044 (H-G3, vaccinated with the higher dose), respectively.

Hare #010 (H-G4) did not show seroconversion after the first challenge with 100 ffu, but had a small increase in RI10 after the second inoculation to 13.32. LeHV-5 was neither detected in blood cells nor skin samples, suggesting the absence of latency or active replication in any of the hares, ruling out the LeHV-5 contribution to the clinical picture and immune response.

In the rabbits, he MYXV antibodies estimated by iELISA at the death (for R-G1) or fifteen days after challenge (R-G2 and R-G3) revealed RI10 lower than 2.0 for the #449, #451, #000 and #001 (R-G1, nonvaccinated); 21.79, 4.31 and 5.71 for #442, #444, and #445 (R-G2, vaccinated with Mixohipra-H); and 33.02, 23.44, 24.48 for #446, #447 and #448 (R-G3, vaccinated with Myxo-RHD PLUS), respectively.

### 3.4. Virus Presence in Drinking Water and Faeces

During the two vaccination trials, no MYXV-DNA was detectable in the drinking water or the hares’ faeces prior challenge.

MYXV-DNA was detected in faeces of hare #231 (H-G1), seven days after virus challenge, four days before the first signs of disease were noticed. For the remaining hares, virus shedding coincided with the appearance of the first clinical signs or appeared 3 to 4 days later, around day 15 or 16 after the virus challenge. The maximum viral load found on faeces was 1.10 × 10^9^ DNA copies/mg and the average value was 4.00 × 10^7^ DNA copies/mg, considering only the DNA-positive samples. The virus was no longer detected in faeces after the animals recovered clinically (clinical signs disappeared) or, in some cases, 48 h before the disappearance of signs of disease.

The type of drinking fountain used for hares consisted of a small shell-shaped reservoir containing a limited volume of water (about 5 mL), from which water was sampled. In hares that developed erosive lesions in the oral mucosa (#231, #232, #233, #042 and #076) the virus was detected in water samples by qPCR. The average viral load in the DNA-positive drinking water samples was 5.00 × 10^6^ DNA copies/mL with a minimum of 6.32 × 10^3^ and a maximum of 4.73 × 10^7^ DNA copies/mL. For one drinking water sample (with a viral load of 1.30 × 10^7^), it was possible to isolate the virus in RK13 cells.

The faeces analyses were not performed in the rabbit trial due to the characteristics of the cages that do not allow the separation of the faeces from each animal. Likewise, the rabbits’ pacifier drinkers do not allow the analysis of the drinking water, and therefore this analysis was not performed.

### 3.5. Necropsy, Histopathology and Virus Loads in Tissues

The necropsy and histopathology data of hares from group H-G1 (#231, #232, #233), H-G2 (#076 and #078) and H-G3 (#042) revealed the expected lesions found in naturally infected animals by ha-MYXV [3,19,20] namely the oedema of eyelids and ano-genital, the production of myxoid tissue and the secondary bacterial infection. No remarkable histopathological changes were observed in animals #043 and #044 (H-G3) artificially immunized by the high dose vaccine, or in #10 (H-G4) naturally immunized.

Rabbits #449, #451, #000, #001 (Table 5) revealed the expected lesions found in naturally infected rabbits by the ha-MYXV [7,8], namely eyelid, ano-genital and alveolar oedemas.

Organs sampled but without description in Table 5 were considered without relevant changes. No myxoma lesions (also called “pseudotumours” or “tumour-like lesions”) were observed in the skin of any animal (rabbit and hares) during the clinical course of the disease or after death, not even in the virus inoculation zone.

No pathological findings in necropsy and histopathology were found in hares #043 (H-G3, vaccinated with high dose) and #010 (H-G4, naturally immunized) and all the vaccinated rabbits: #442, #444 and #445 (R-G2, vaccinated with M-H) and #446, #447 and #448 (R-G3, vaccinated with Myxo-RHD PLUS), and they were also negative to bacteriological and parasitological analyses (not included in Table 5). Hare #044 (H-G3) only presented mild scar on the eyelids.

Several pathogenic bacteria were found in the animals included in this study (Table 5). In general, mild infections by *Eimeria* species were found in sick animals, and these parasites were very frequently also found in wild animals that died from myxomatosis or in healthy animals [20]. Despite the infection, no signs compatible with enteritis were detected at necropsy and no diarrhoea events were registered during the entire course of the trial in these animals.

The viral loads in the different tissues (Table 6) were determined by qPCR [16]. The highest viral loads were registered in the eyelids (mean of all positive hares of 1.10 × 10^10^ ± 1.02 × 10^10^ DNA copies/mg tissue and mean of all positive rabbits of 4.79 × 10^10^ DNA copies/mg tissue), lips (mean of all positive hares of 9.30 × 10^9^ ± 1.60 × 10^10^ DNA copies/mg tissue and mean of all positive rabbits of 1.22 × 10^9^ ± 8.88 × 10^8^ DNA copies/mg tissue) and genitalia (mean of all positive hares of 1.65 × 10^10^ ± 2.52 × 10^10^ DNA copies/mg tissue and mean of all positive rabbits of 2.53 × 10^10^ ± 2.43 × 10^10^ DNA copies/mg tissue).

Overall, no virus was detected in vaccinated rabbits independently of the vaccine used, but was detected in all non-vaccinated rabbits. In all hares submitted to virus challenge, virus was detected in the tissues except for 2 out of the three hares vaccinated with 10× M-FSA vaccine, which seroconverted.

## 4. Discussion

The objectives established for this study were substantially different for the two-animal species. For the Iberian hare, the main objective was to investigate if commercially available MYXV vaccines for use in rabbits might constitute a prophylactic tool for hares until a hare specific vaccine is available. The objectives for the wild rabbit were to prove the efficacy of commercial rabbit vaccines in wild rabbits against the recently emerged ha-MYXV (either isolated from Iberian hare or wild rabbit) and to investigate the susceptibility of wild rabbits to the ha-MYXV isolated from the Iberian hare.

Several vaccines against myxomatosis are currently available and can show protection when used properly in wild rabbits [21]. However, there are some disagreements in determining the effectiveness of vaccination campaigns in the wild as a management measure [22]. Vaccination campaigns of wild rabbits against myxoma virus are usually “blind”, and non-systematic: vaccines are often administrated to the animals regardless of their sex, age or serological status [23].

The commercial myxomatosis vaccines contain live attenuated virus because multiplication of the virus, despite being limited, is important for the induction of a robust immune response, comprising also cellular immunity which is key for protection to myxomatosis [24]. As commercial vaccines against myxomatosis contain classic MYXV strains (e.g., VMI 30 strain) or a Shope fibroma virus strain, which are different from ha-MYXV to which the Iberian hare is highly susceptible, low efficacy of commercial rabbit vaccines is expected, given the absence of vaccine virus multiplication in hare cell cultures (personal communication).

The greatest limitation of this study was imposed by the small number of hares and wild rabbits included in the trial (three per group), intended to reduce to a minimum the number of animals, given the current critical situation of these populations and ethical issues. Despite this assumed constraint, our results showed that hares vaccinated with two commercially available vaccines (Mixohipra-FSA and Mixohipra-H) in the conditions recommended for rabbits, did not seroconvert robustly. Nor did the vaccinated hares gain protection against challenge with ha-MYXV, even when a very low dose of challenge virus (100 ffu) was used, close to that used in previous rabbit studies [25,26]. In fact, with this standard vaccination protocol, all vaccinated hares developed severe disease after challenge, similarly to the non-vaccinated controls.

Interestingly, 2 out of the 3 hares vaccinated with a 10-fold higher dose as recommended for domestic rabbits of Mixohipra-FSA, and a 10-fold higher dose of Mixohipra-H 21 days later, induced a satisfactory humoral response against MYXV. However, there was no remarkable change in antibody titres after the Mixohipra-H boost, which seems to indicate that vaccination with Mixohipra-FSA alone might be sufficient to induce protective immunity in hares. It is worth noting that the higher doses were not accompanied by any general or local (inoculation site) reactions. Furthermore, the haematological and biochemical data of the groups of hares vaccinated with the lower doses revealed no differences in globulins, contrasting with the decrease in the albumin/globulin ratio, in the case of hare #043 and hare #044 (H-G3), vaccinated with the 10-fold vaccine doses.

This difference in humoral response against these two vaccines, with a higher response against a heterologous vaccine, may be explained, among other reasons, by the fact that Mixohipra-FSA is an adjuvanted (attapulgite) vaccine and Mixohipra-H does not contain adjuvant, according to the manufacturer’s documentation. However, further studies are needed to evaluate this hypothesis, namely by testing seroconversion after two successive administrations of Mixohipra-H, without a previously inoculation of Mixohipra-FSA.

The efficient containment of the Iberian hare for clinical evaluation and sampling is extremely difficult and represents a high risk of self-injury (vertebral fracture) due to sudden movements. Therefore, the subcutaneous route was chosen for all administrations considering that intradermal administration implies the total immobilization of the animal for a few minutes to allow the administration of an invariable dose in all animals. Interestingly, hares #042 and #233 did not produce any disease after the first challenge trial (100 ffu), neither seroconverted after vaccination (#042) nor virus challenge (#042 and #233), showing a potential difference in the genetic susceptibility of some animals. No signs of cervical lymph node reaction were detected after the virus challenge. This can also mean that the subcutaneous route is not the ideal primary site of Myxoma virus multiplication or antigen presentation. Several studies have shown the lower effectiveness of the subcutaneous route in inducing immunity compared with the intradermal route [27,28]. The intradermal route allows a longer contact between the antigen and the antigen-presenting cells with a high number of dendritic cells in the derma compared with the subcutaneous tissue [29]. When Myxoma virus is inoculated intradermically, it can enter directly by lymphatic vessels for transport to antigen-presenting cells in the lymph nodes [30]. Considering the specificity of MYXV to epithelial cells, the delivery to the epidermis or dermis may result in superior and quick immune responses when compared to muscle and subcutaneous tissues [28]. This explanation can be also applied to the inoculation of virus during the challenge. Dalton et al. [27] found seropositivity of only 16.6–54% of rabbits after subcutaneous vaccination with a delay in seroconversion of these animals. However, in these two hares (#233 and #042), the second inoculation with 1000 ffu induced the typically fatal disease. This can mean that 100 ffu might be a low dose for a viral challenge by subcutaneous route, and is why a dose of 1000 ffu was used in the challenge carried out on rabbits, bearing in mind that this test was made after that of hares. Studies carried out in rabbits used different viral doses, namely 2 × 10^5.4^ TCID50 (around 1.4 × 10^5.4^ ffu) [31], unknown viral load [32] inoculated subcutaneously, or a dose of around 10^2^ to 10^4^ [33,34,35,36,37,38,39] inoculated intradermically.

In wild rabbits, both Mixohipra-H and Nobivac Myxo-RHD PLUS vaccines, administered in the dose recommended for domestic rabbits, induced a humoral response and completely protected the animals from experimental infection, as none of the rabbits’ showed signs of disease after challenge. The wild rabbit is the same species as experimental, pet and industrial rabbits so it is expected that a similar response against the vaccination is developed, allowing the vaccine virus infection and replication in this species. According to the previous statement, the humoral and cellular response against the vaccine strain is expected to be protective against the naturally recombinant virus, taking into account that most of (all) the antigenic epitopes of classic MYXV are conserved in this recombinant virus.

Non-vaccinated rabbits succumbed to inoculation with ha-MYXV either isolated from Iberian hare (38455PT18) or wild rabbit (20545PT20), demonstrating, for the first time, the susceptibility of *Oryctolagus cuniculus* to the recombinant virus directly isolated from Iberian hare. Although these two strains (38455PT18 and 20545PT20) are still being fully characterized, the disruptive insertion of the M009L gene is conserved in both as well as the insertion affecting the M152R gene (Serp-3), a known virulence factor of MYXV [40].

Despite some differences were observed between the animals that developed severe myxomatosis (Table 3, Table 4 and Table 5), it was not possible to establish any clinical or lesional pattern between the hares of the different groups due to the small size of our sample, as it is likely that the differences found are due to natural inter-individual variability. However, the differences between the anatomopathological patterns found in hares and in rabbits was evident, probably as a result of the more rapid clinical evolution in the latter.

The macroscopic and histopathologic lesions found in hares and rabbits were similar to those described previously [1,3,7,8,19].

Myxomas (tumour-like lesions) were neither found in the skin of the hare or rabbit used in the three studies, nor in wild naturally infected with ha-MYXV [7], although they have already been found in domestic rabbits infected with ha-MYXV [8]. Furthermore, myxomas are not always present in wild hares found dead with ha-MYXV, being only present in around 30% of hares [6,7,8,20]. According to classic myxoma virus virulence grade classification [18,41,42], mortality of 100% in seronegative hares would correspond to Grade I viruses. However, in this assay, the average survival time was of 17.8 ± 8.5 days after symptoms onset and not ≤13 days as in rabbits infected in Grade I viruses, suggesting that this classification is not appropriate for the Iberian hare. More studies are therefore needed to understand these differences.

All the immunized hares and rabbits showed a moderate to high increase of the antibody titres after the challenge, showing a non-sterilizing immunity. Interestingly, rabbit #10, which acquired immunity by recovering from a natural infection, showed no response after inoculation of 100 ffu of virus, with a small increase in antibodies after the second inoculation.

PCR based viral quantification revealed loads compatible with what was previously described [3,7,8]. As expected [7,8,20], a higher viral load was found in the skin (eyelid and lips) and external genitalia, of both hares and rabbits. However, a variable load of virus was found in various organs, proving systemic dissemination. Relatively high virus loads were also found in the central nervous system (brain and spinal cord), as reported previously for the classic virus strains [18].

The viral loads of ha-MYXV found in hares and rabbits were similar, regarding titre and distribution in different organs, suggesting similar pathophysiology and organic distribution of the virus in both species (Table 6), and independent from the dose of virus inoculated (100 or 1000 ffu). However, since the animals were not euthanized and did not die on the same day, the different periods of viral replication hamper further conclusions.

However, an important difference to highlight is the higher viral load (10–100× higher titres) in the lungs of rabbits compared to hares, which may be related to the fact that the clinical course in rabbits was more acute. This was true even when comparing the rabbits with the two hares (#042 and #233) infected with 1000 ffu.

This difference was particularly notable in animals inoculated with wild rabbit strain (#449 and #451, inoculated with 20545PT20), whose external lesions were scarcely evident, and that succumbed as a result of pulmonary alterations and accumulation of pleural effusion. These two animals died 12 days after virus inoculation and 4 days after the onset of symptoms. According to classic myxoma virus virulence grade classifications [18,41,42], these two virus strains can be classified as a Grade 1 (the highest virulence).

No particularly important bacteriological pathology was found in hares’ histopathology, and opportunistic bacteria were generally found, probably as a result of immunosuppression caused by the myxoma virus [43]. The bacteriological findings were surprising, revealing the presence of several bacteria species in leporids for the first time, namely *Stenotrophomonas maltophilia*, *Enterococcus gallinarum* and *Vibrio vulnificus*, and confirming that secondary bacterial infections may have contributed significantly to the dead of the animals (Table 5). *Staphylococcus equorum* was also reported in nasal samples from wild rabbits in Azores, Portugal [44], but was never reported in hares, including the Iberian hare. *Stenotrophomonas maltophilia* was never reported in rabbits or hares, being an aerobic, non-fermentative, Gram-negative bacterium, uncommon and difficult to treat in humans [45]. *Enterococcus gallinarum* was also never reported in hares or rabbits, also being of zoonotic importance [46,47]. The *Vibrio vulnificus* is a multi-host opportunistic bacteria [48] never reported in leporids. This bacteria leads to human mortality rates of 50% by sepsis and of 17% due to wound infection [49]. Besides infection is rare, this species is responsible for the most deaths caused by Vibrios [49]. The hare species are known for their reservoir potential for many other emerging or re-emerging pathogens of public health importance, namely *Yersinia* spp., *Brucella* spp., and *Francisella tularensis* (reviewed in [50]).

The following conclusions can be taken from this study: (i) the Iberian hare is not protected from mortality due to myxomatosis by Mixohipra-FSA and/or Mixohipra-H according to the dose used in domestic rabbits, (ii) it is possible to protect the Iberian hare from mortality due to myxomatosis using a higher dose of Mixohipra-FSA, (iii) the commercial vaccines Mixohipra-H and Nobivac Myxo-RHD PLUS protect the wild rabbit effectively against natural recombinant myxoma virus strains and (iv) the wild rabbit is susceptible to ha-MYXV directly isolated either from the wild rabbit or the Iberian hare.

This finding indicates that wild rabbits may contribute to the spread of ha-MYXV in hares. As two commercial myxoma vaccines (Mixohipra-FSA and Mixohipra-H) showed no efficacy in hares when using commercially recommended dosages, it is urgent to develop a robust vaccine for the Iberian hare or to investigate vaccine efficacy in hares of other commercial myxoma vaccines.

## Figures and Tables

**Table 1:** Serological responses of hares (study 1) after vaccination and before challenge. Data were measured by indirect ELISA (RI10), immunofluorescence titration (IFT = titre in log2) and seroneutralization (SNT = titre).

				Humoral Response						
Group	Hare ID	Vaccine	Technique	Day 0	Day 7	Day 21	Vaccine	Day 28	Day 42	Day 72
H-G1	#231	Not vac	iELISA	0.5	0.23	0.16	Not vac	0.13	0.19	0.23
			IFT	<2	<2	<2		<2	<2	<2
			SNT	<1/4	<1/4	<1/4		<1/4	<1/4	<1/4
	#232	Not vac	iELISA	0.35	0.16	0.39	Not vac	0.29	0.15	0.28
			IFT	<2	<2	<2		<2	<2	<2
			SNT	<1/4	<1/4	<1/4		<1/4	<1/4	<1/4
	#233	Not vac	iELISA	0.63	0.47	0.42	Not vac	0.22	0.22	0.08
			IFT	<2	<2	<2		<2	<2	<2
			SNT	<1/4	<1/4	<1/4		<1/4	<1/4	<1/4
H-G2	#076	1× M-FSA	iELISA	0.9	1.01	0.84	1× M-H	0.59	0.59	0.18
			IFT	<2	3	3.5		4	4	4
			SNT	<1/4	<1/4	<1/4		<1/4	<1/4	<1/4
	#077	1× M-FSA	iELISA	0.31	0.81	1.55	1× M-H	2.45	2.81	-
			IFT	<2	<2	4		4	5.5	
			SNT	<1/4	<1/4	<1/4		1/8	1/8	
	#078	1× M-FSA	iELISA	0.96	0.49	0.4	1× M-H	0.36	0.36	0.12
			IFT	<2	<2	3		3	3	3
			SNT	<1/4	<1/4	<1/4		<1/4	<1/4	<1/4
H-G3	#042	10× M-FSA	iELISA	0.6	0.36	0.25	10× M-H	0.24	0.18	0.03
			IFT	<2	<2	3		3	3	3
			SNT	<1/4	<1/4	<1/4		<1/4	<1/4	<1/4
	#043	10× M-FSA	iELISA	0.79	0.62	5.55	10× M-H	5.05	5.5	5.8
			IFT	<2	4.5	6.5		7	7	7.5
			SNT	<1/4	<1/4	1/16		1/16–1/32	1/16–1/32	1/16–1/32
	#044	10× M-FSA	iELISA	0.75	0.36	5.57	10× M-H	6.87	6.7	7.1
			IFT	<2	3.5	7		7.5	7	8.5
			SNT	<1/4	<1/4	1/16		1/16–1/32	1/16–1/32	1/16–1/32
H-G4	#10	Natural immunity	iELISA			7.09	Natural immunity	7.1	7.19	7.28
			IFT			9		9	9	9
			SNT			1/128		1/128	1/128	1/128

1× M-FSA, vaccinated with 2.90 × 10^4^ ffu of Mixohipra-FSA; 10× M-FSA, vaccinated with 2.90 × 10^5^ ffu dose of Mixohipra-FSA; 1× M-H, vaccinated with 1.95 × 10^4^ ffu dose of Mixohipra-H; 10× M-H, vaccinated with 1.95 × 10^5^ ffu dose of Mixohipra-H; Not vac, not vaccinated.

**Table 2:** Serological responses of rabbits (study 2) after vaccination and before challenge. Data were measured by indirect ELISA (RI10), immunofluorescence titration (IFT = titre in log2) and seroneutralization (SNT = titre). RI10.

				Humoral Response				
Group	Rabbits ID	Vaccine	Technique	Day 0	Day 7	Day 21	Day 28	Day 35
R-G1	#449	Not vac	iELISA	0.05	0.06	0.07	0.06	0.02
			IFT	<2	<2	<2	<2	<2
			SNT	<1/4	<1/4	<1/4	<1/4	<1/4
	#451	Not vac	iELISA	0.22	0.01	0.1	0.27	0.2
			IFT	<2	<2	<2	<2	<2
			SNT	<1/4	<1/4	<1/4	<1/4	<1/4
	#000	Not vac	iELISA	0.13	0.09	0.15	0.18	0.31
			IFT	<2	<2	<2	<2	<2
			SNT	<1/4	<1/4	<1/4	<1/4	<1/4
	#001	Not vac	iELISA	0.32	0.27	0.38	0.21	0.14
			IFT	<2	<2	<2	<2	<2
			SNT	<1/4	<1/4	<1/4	<1/4	<1/4
R-G2	#442	1× M-H	iELISA	0.01	0.05	1.79	2.33	4.11
			IFT	<2	4	6	7.5	9
			SNT	<1/4	<1/4	<1/4	1/8	1/32
	#444	1× M-H	iELISA	0.04	0.18	1.1	1.04	1.72
			IFT	<2	2	5.5	6	7
			SNT	<1/4	<1/4	<1/4	1/4	1/8
	#445	1× M-H	iELISA	0.1	0.29	0.83	1.04	2.15
			IFT	<2	3	<2	6	8.5
			SNT	<1/4	<1/4	<1/4	1/4	1/8
R-G3	#446	1× M-RHD	iELISA	0.31	0.36	2.3	3.05	4.78
			IFT	<2	3.5	6.5	8	9
			SNT	<1/4	<1/4	1/8	1/16	1/64
	#447	1× M-RHD	iELISA	0.35	0.21	5.32	5.42	5.39
			IFT	<2	4.5	9.5	10	10
			SNT	<1/4	1/4	1/128	1/128	1/128
	#448	1× M-RHD	iELISA	0.26	0.51	1.21	1.09	2.15
			IFT	<2	4	5.5	6	7
			SNT	<1/4	<1/4	1/4	1/8	1/8–1/16

1× M-H—vaccinated with 1.95 × 10^4^ ffu dose of Mixohipra-H; 1×-M-RHD—vaccinated with 1 dose of Myxo RHD-PLUS; Not vac—not vaccinated.

**Table 3:** Clinical data of Iberian hares after challenge with ha-MYXV (38455PT18).

			Days after Virus Challenge						
Group	Animal ID	0	1–9	10–15	16–20	21–25	26–30	31–40	D41–D50
H-G1	Hare #231 Not vac	Virus challenge with 1 mL (100 ffu/mL) of ha-MYXV (38455PT18)	N	D11-very mild eyelid oedema. <25% of eye closure. D13—mild eyelid oedema. <25% of eye closure. D14—moderate eyelid oedema. 50% of eye closure.	D16 marked eyelid oedema. 50–75% of eye closure.	D25 marked eyelid oedema. >75% of eye closure.	D30-marked eyelid and anogenital oedema. 100% of eye closure. Anorexia and dyspnea. Euthanasia.		
	Hare #232 Not vac		N	D12—mild eyelid oedema. <25% of eye closure. D14—moderate eyelid oedema. 25–50% of eye closure.	D18 marked eyelid oedema. 50–75% of eye closure.	D25 marked eyelid oedema. >75% of eye closure.	D30 marked eyelid and anogenital oedema. 100% of eye closure. Anorexia and dyspnea. Euthanasia.		
	Hare #233 Not vac		N	N	N	N	D30—Second virus challenge with 1000 ffu of ha-MXYV (38455PT18), day 102 in Figure 1.	D40—very mild eyelid oedema. <25% of eye closure. D42—mild eyelid oedema. <25% of eye closure.	D45—Marked eyelid, foreskin and anogenital oedema. 50–75% of eye closure. Anorexia. D50-Death
H-G2	Hare #076 1× M-FSA 1× M-H		N		D16—very mild eyelid oedema. <25% of eye closure. D18—very mild eyelid oedema. <25% of eye closure.	D20—mild eyelid oedema. <25% of eye closure.	D20—moderate eyelid oedema. 25–50% of eye closure. D30—marked eyelid oedema. 50–75% of eye closure.	D40—marked eyelid oedema. >75% of eye closure.	D48—marked eyelid and foreskin oedema. >75% of eye closure. Anorexia and dyspnoea. Euthanasia.
	#078 Hare 1× M-FSA 1× M-H		N	D12—very mild eyelid oedema. <25% of eye closure.	D16—mild eyelid oedema. <25% of eye closure.	D20—mild eyelid oedema. <25% of eye closure. Death			
H-G3	Hare #042 10× M-FSA 10× M-H		N	N	N	N	D30—Second virus challenge with 1000 ffu of ha-MXYV (38455PT18), day 102 in Figure 1.	D40—very mild eyelid oedema. <25% of eye closure. D45—mild eyelid oedema. <25% of eye closure.	D50—mild eyelid oedema. <25% of eye closure D60—moderate eyelid, testis and foreskin oedema. 25–50% of eye closure. Anorexia. Death.
	Hare #043 10× M-FSA 10× M-H		N	D12-very mild eyelid oedema. <25% of eye closure.	D16—Left l ower eyelid erythema.	D25—N	N	N	N
	Hare #044 10× M-FSA 10× M-H		D7—mild eyelid oedema. <25% of eye closure.	D12—mild eyelid oedema. <25% of eye closure.	D14-D20—moderate eyelid oedema. 25–50% of eye closure.	D21—Beginning of crusting and improvement of eye opening.	D26—N, in addition to scars on the eyelids.		
H-G4	Hare #10 Natural immunity		N	N	N	N	D30-Second virus challenge with 1000 ffu of 38455PT18.	N	N

1× M-FSA,—vaccinated with 2.90 × 10^4^ ffu dose of Mixohipra-FSA; 10× M-FSA—vaccinated with 2.90 × 10^5^ ffu dose of Mixohipra-FSA; 1× M-H—vaccinated with 1.95 × 10^4^ ffu dose of Mixohipra-H; 10× M-H—vaccinated with 1.95 × 10^5^ ffu dose of Mixohipra-H, Not vac-Not vaccinated; D-day, N—no disease signs.

**Table 4:** Clinical data of wild rabbits after challenge with ha-MYXV isolated from wild rabbit (20545PT20) or Iberian hare (38455PT18).

			Days after Virus Challenge						
Group	Animal ID	0	1–9	10–15	16–20	21–25	26–30	31–40	D41–D50
RG-1 Not vac	Rabbit #000	Virus challenge of 1000 ffu of ha-MYXV (38455PT18), day 65 in Figure 1.	D4—very mild eyelid oedema. No genital changes. D8—moderate eyelid and genital oedema. 50–75% of eye closure.	D11—Marked eyelid, foreskin and genital oedema. >75% of eye closure Anorexia. Death.					
	Rabbit #001		D5—very mild eyelid and genital oedema. D8—moderate eyelid and genital oedema. 50–75% of eye closure.	D11—Marked eyelid, foreskin and genital oedema. >75% of eye closure Anorexia. Death.					
	Rabbit #449	Virus challenge of 1000 ffu of ha-MYXV (20545PT20), day 35 in Figure 1.	D8—very mild eyelid oedema. <25% of eye closure. No genital changes.	D12—Very mild eyelid and foreskin oedema. <25% of eye closure. No genital oedema. Death.					
	Rabbit #451		D8—very mild eyelid oedema. <25% of eye closure. No genital changes.	D12—Very mild eyelid and foreskin oedema. <25% of eye closure. No genital oedema Death.					
RG-2 1× M-H	Rabbit #442		N	N	N	N	N	N	N
	Rabbit #444		N	N	N	N	N	N	N
	Rabbit #445		N	N	N	N	N	N	N
RG-3 1× M-RHD	Rabbit #446		N	N	N	N	N	N	N
	Rabbit #447		N	N	N	N	N	N	N
	Rabbit #448		N	N	N	N	N	N	N

1× M-H—vaccinated with 1.95 × 10^4^ ffu dose of Mixohipra-H; 1× M-RHD—vaccinated with 1 dose of Myxo-RHD PLUS; Not vac-not vaccinated; D-day, N—no disease signs.

**Table 5:** Necropsy, bacteriological and parasitological data from hares and rabbits.

Group	Animal ID	Challenge	Pathological Examination					Bacteriological Examination from Liver, Spleen and Lungs	Parasitological Examination
			Eyelid/Ano-Genitalia/Lip	Liver	Spleen	Lungs	Kidney/Other		
H-G1	Hare #231	100 ffu of ha-MYXV isolated from hare (38455PT18)	Macroscopic: Marked eyelid, lips, genitalia and anus oedema. Thickening and congestion of the scrotal sac wall with necrosis. Accumulation of fibrin within the scrotal pouches. Congestion of the testicles. Microscopic: Eyelid with necrosis of the epidermis and conjunctiva with bacterial infiltration. Scrotum with oedema at the dermo-epidermal junction with detachment of the epidermis. Lip with necropurulent dermatitis and presence of extensive bacterial clusters. Testis with absence of germ cells, persisting only Sertoli cells. Epididymis with necrosis of the lining epithelium and accumulation of necrotic cells in the lumen of the ducts. *Vas deferens* with accumulation of myxoid tissue underlying the lamina propria.	Moderate periportal infiltration by mononuclear cells	N	N	Kidney with perivascular lymphocytic infiltration. Empty stomach.	*Escherichia coli* and *Stenotrophomonas maltophilia*	Mild infection by *Eimeria* spp.
	Hare #232		Macroscopic: Marked eyelid, lips, genitalia and anus oedema. Microscopic: Eyelid with epidermal hyperplasia and extensive proliferation of myxoid tissue in depth to the conjunctiva. Lips with epidermal hyperplasia, ballooning degeneration of keratinocytes, intense production of myxoid tissue, infiltration of muscle tissue by mononuclear cells and heterophils. Prepuce with extensive and severe necropurulent dermatitis with strong bacterial infiltration, myxoid tissue in the dermis, diffuse infiltration by heterophils in depth, necropurulent foci.	Cellular infiltration, namely by heterophils in the porta spaces and occasionally in the sinusoid capillaries.	Marked depletion of lymphocytes.	Multifocal purulent alveolitis.	Kidney with perivascular infiltration by mononuclear cells. Empty stomach.	*Stenotrophomonas maltophilia, Staphylococcus equorum and Staphylococcus xylosus*	Mild infection by *Eimeria* spp.
	Hare #233	1000 ffu of ha-MYXV isolated from hare (38455PT18)	Macroscopic: Nodular thickening on the eyelids and lips and oedema of the prepuce. Microscopic: Eyelid with epidermal hyperplasia and vacuolization of keratinocytes. Proliferation of myxoid tissue in the dermis. Diffuse microhaemorrhages and foci of infiltration by heterophils. Conjunctival epithelial hyperplasia and underlying stromal oedema. Lips with epithelial hyperplasia and marked vacuolization of keratinocytes with proliferation of myxoid tissue in the dermis and intense infiltration by heterophils. Foreskin with thick necropurulent exudate and extensive haemorrhages, presence of myxoid tissue and infiltration by heterophils.	N	Marked depletion of lymphocytes.	Lung collapse.	Empty stomach.	*Escherichia coli* and *Enterobacter sakazakii*	Mild infection by *Eimeria leporis*.
H-G2	Hare #076	100 ffu of ha-MYXV isolated from hare (38455PT18)	Macroscopic: Marked oedema of the prepuce, eyelids and lips. Empty stomach. Microscopic: Eyelid with dermal oedema. Lips with foci of epithelial hyperplasia and vacuolization of keratinocytes. Foreskin with marked oedema of the dermis.	Liver with periportal infiltration by mononuclear cells and heterophils in sinusoid capillaries.	Mild depletion of lymphocytes.	Congestion.		*Escherichia coli*	N
	Hare #078		Macroscopic: Marked oedema of the foreskin, eyelids and lips. Presence of small ulcers on the lips. Microscopic: Lips with foci of epithelial hyperplasia and vacuolization of keratinocytes. Foreskin with marked oedema of the dermis. Epididymis with oedema and extensive haemorrhages in the basement membrane zone. Hyperplasia of the duct lining epithelium and desquamation of the ductal epithelium. Infiltration by heterophils. Eyelid with dermal oedema.	Liver with periportal infiltration, mainly by mononuclear cells and heterophils in sinusoid capillaries.	Marked depletion of lymphocytes.	Congestion.	Kidney with the presence, mostly perivascular, of small clusters of lymphoid cells. Empty stomach.	*Enterococcus gallinarum*	Marked infection by *Eimeria* spp.
H-G3	Hare #042	1000 ffu of ha-MYXV isolated from hare (38455PT18)	Macroscopic: Nodular thickening and moderate oedema and nodular thickening of eyelids, nose, lips and testis. Oedema of the extremities of the hind limbs. Microscopic: Eyelid with epidermal hyperplasia and vacuolization of keratinocytes. Moderate proliferation of myxoid tissue in the dermis. Lips with necropurulent lesions, moderate epithelial hyperplasia and vacuolization of keratinocytes with proliferation of myxoid tissue in the dermis and infiltration by heterophils. Foreskin with moderate presence of myxoid tissue and infiltration by heterophils.	Congestion.	Marked depletion of lymphocytes.	*Congestion.*	Empty stomach.	*Vibrio vulnificus*	N
	Hare #044	100 ffu of ha-MYXV isolated from hare (38455PT18)	Macroscopic: Mild scar on the eyelids.	N	N	N	N	N	N
R-G1	Rabbit #449 and Rabbit #451	1000 ffu of ha-MYXV isolated from rabbit (20545PT20)	Macroscopic: Very mild oedema of the eyelids and lips. Presence of extensive pleural effusion with fibrin. Microscopic: Eyelid with mild oedema of the deep dermis.	Congestion of Liver (#449).	Spleen with total necrosis of lymphoid follicles with only a very congested red pulp visible.	Lung congestion and oedema. Pleural efusion. Necrosis of peribronchial lymphoid tissue.	Full stomach.	N	N
	Rabbit #000 and Rabbit #001	1000 ffu of ha-MYXV isolated from hare (38455PT18)	Macroscopic: Marked swelling of the eyelids, lips and external genitalia. Presence of purulent mucous exudate on the eyelids. Reduced content in the stomach. Microscopic: Eyelid with marked oedema, small foci of myxoid tissue in the dermis, purulent conjunctivitis of bacterial etiology. Lip with oedema and small foci of myxoid tissue.	N	Spleen with congestion and necrosis of lymphoid follicles.	N	N	N	Medium infection with *Eimeria perforans*.

N—no remarkable changes or negative to bacterial or parasitological analysis.

**Table 6:** Viral loads in the hare and rabbit tissues after challenge.

ID	Vaccination Data		Viral Loads in Different Tissues (DNA Copies/mg Tissue)												
	1st	2nd	Liver and Spleen	Lung	Duodedum	Kidney	Eyelid	Lip	Genitalia	Urine	Seminal Vesicle	Faeces	Brain	Bone Marrow	Spinal Cord
Hares															
#231	Not vac	Not vac	1.60 × 10^7^	1.47 × 10^8^	5.76 × 10^6^	1.71 × 10^7^	2.88 × 10^9^	1.75 × 10^9^	2.23 × 10^7^	7.44 × 10^6^	1.78 × 10^9^	3.98 × 10^8^	4.05 × 10^6^	1.31 × 10^6^	1.53 × 10^6^
#232	Not vac	Not vac	1.10 × 10^8^	1.23 × 10^8^	3.66 × 10^7^	3.15 × 10^9^	1.24 × 10^10^	7.47 × 10^9^	1.24 × 10^10^	5.47 × 10^3^	5.42 × 10^6^	1.83 × 10^6^	4.95 × 10^5^	3.70 × 10^5^	5.45 × 10^6^
#233	Not vac	Not vac	6.73 × 10^8^	6.28 × 10^8^	4.03 × 10^6^	4.39 × 10^8^	1.34 × 10^10^	3.88 × 10^9^	5.33 × 10^10^	NT	5.37 × 10^5^	1.52 × 10^8^	2.29 × 10^8^	6.83 × 10^4^	9.91 × 10^4^
#076	1× M-FSA	1× M-H	2.55 × 10^5^	2.33 × 10^5^	5.59 × 10^4^	1.85 × 10^6^	1.34 × 10^7^	1.11 × 10^6^	2.08 × 10^8^	2.16 × 10^5^	ND	1.16 × 10^5^	ND	3.11 × 10^3^	ND
#077	1× M-FSA	1× M-H	ND	ND	ND	ND	ND	ND	ND	ND	ND	ND	ND	ND	ND
			This animal was not submitted to challenge but was analysed to MYXV to eliminate as the cause of death												
#078	1× M-FSA	1× M-H	1.17 × 10^8^	4.39 × 10^8^	7.20 × 10^7^	2.22 × 10^8^	2.89 × 10^10^	4.16 × 10^10^	5.40 × 10^9^	NT	1.36 × 10^5^	1.50 × 10^9^	6.26 × 10^6^	3.22 × 10^4^	8.54 × 10^6^
#042	10× M-FSA	10× M-H	2.90 × 10^7^	3.06 × 10^7^	8.72 × 10^6^	1.50 × 10^7^	8.46 × 10^9^	1.08 × 10^9^	7.73 × 10^9^	NT	NT	7.51 × 10^8^	7.42 × 10^4^	9.25 × 10^4^	2.36 × 10^5^
#043	10× M-FSA	10× M-H	ND	ND	ND	ND	ND	ND	ND	ND	ND	ND	ND	ND	ND
#044	10× M-FSA	10× M-H	ND	ND	ND	ND	ND	ND	ND	ND	ND	ND	ND	ND	ND
#10	Natural immunity		ND	ND	ND	ND	ND	ND	ND	ND	ND	ND	ND	ND	ND
Rabbits															
#442	1× M-H		ND	ND	ND	ND	ND	ND	ND	ND	ND	ND	ND	ND	ND
#444			ND	ND	ND	ND	ND	ND	ND	ND	ND	ND	ND	ND	ND
#445			ND	ND	ND	ND	ND	ND	ND	ND	ND	ND	ND	ND	ND
#446	1× M-RHD		ND	ND	ND	ND	ND	ND	ND	ND	ND	ND	ND	ND	ND
#447			ND	ND	ND	ND	ND	ND	ND	ND	ND	ND	ND	ND	ND
#448			ND	ND	ND	ND	ND	ND	ND	ND	ND	ND	ND	ND	ND
#449	Not vac		2.30 × 10^10^	2.03 × 10^10^	7.16 × 10^8^	9.97 × 10^8^	1.29 × 10^10^	1.36 × 10^9^	4.19 × 10^10^	2.84 × 10^7^	ND	2.22 × 10^4^	ND	1.02 × 10^2^	ND
#451			1.24 × 10^10^	3.48 × 10^10^	1.04 × 10^8^	4.94 × 10^9^	1.87 × 10^10^	9.97 × 10^8^	3.01 × 10^9^	8.72 × 10^6^	NT	1.15 × 10^4^	ND	8.91 × 10^2^	ND
#000	Not vac		9.91 × 10^9^	7.74 × 10^10^	1.25 × 10^8^	1.45 × 10^9^	6.98 × 10^10^	2.33 × 10^9^	6.07 × 10^9^	1.15 × 10^8^	NT	1.24 × 10^5^	3.13 × 10^4^	ND	ND
#001			3.32 × 10^8^	2.11 × 10^9^	2.93 × 10^7^	8.32 × 10^9^	9.02 × 10^10^	1.89 × 10^8^	5.04 × 10^10^	2.04 × 10^6^	NT	2.25 × 10^3^	4.12 × 10^3^	2.15 × 10^0^	ND

1× M-FSA—vaccinated with 2.90 × 10^4^ ffu dose of Mixohipra-FSA; 10× M-FSA—vaccinated with 2.90 × 10^5^ ffu dose of Mixohipra-FSA; 1× M-H—vaccinated with 1.95 × 10^4^ ffu dose of Mixohipra-H; 10× M-H—vaccinated with 1.95 × 10^5^ ffu dose of Mixohipra-, 1×-M-RHD—vaccinated with 1 dose of Myxo RHD-PLUS; Not vac. Not vaccinated; NT—non tested; ND—non detected H—Iberian hare R-wild rabbit.

## Data Availability

All data are presented in this study, there are no additional data.
